# Imaging gene and environmental effects on cerebellum in Attention-Deficit/Hyperactivity Disorder and typical development^☆^

**DOI:** 10.1016/j.nicl.2012.11.010

**Published:** 2012-12-06

**Authors:** Patrick de Zeeuw, Janna van Belle, Sarai van Dijk, Juliette Weusten, Bobby Koeleman, Esther Janson, Herman van Engeland, Sarah Durston

**Affiliations:** a Neuroimaging Lab, Developmental Disorders Unit, Department of Psychiatry, Rudolf Magnus Institute of Neuroscience, University Medical Center Utrecht, Heidelberglaan 100, 3584 CX, Utrecht, The Netherlands; b Department of Medical Genetics, Research Section, Rudolf Magnus Institute of Neuroscience, University Medical Center Utrecht, Heidelberglaan 100, 3584 CX, Utrecht, The Netherlands

**Keywords:** ADHD, Attention-deficit/hyperactivity disorder, CD, Conduct disorder, ODD, Oppositional defiant disorder, SNP, Single nucleotide polymorphism, XKR4, XK-Kell blood group complex subunit-related family, member 4, ADHD, Cerebellum, Birth weight, XKR4, Gene environment interaction

## Abstract

This study investigates the effects of XKR4, a recently identified candidate gene for Attention-Deficit/Hyperactivity Disorder (ADHD), birth weight, and their interaction on brain volume in ADHD. XKR4 is expressed in cerebellum and low birth weight has been associated both with changes in cerebellum and with ADHD, probably due to its relation with prenatal adversity. Anatomical MRI scans were acquired in 58 children with ADHD and 64 typically developing controls and processed to obtain volumes of cerebrum, cerebellum and gray and white matter in each structure. DNA was collected from saliva. Analyses including data on birth weight were conducted in a subset of 37 children with ADHD and 51 controls where these data were retrospectively collected using questionnaires. There was an interaction between genotype and birth weight for cerebellum gray matter volume (*p* = .020). The combination of homozygosity for the G-allele (the allele previously found to be overtransmitted in ADHD) and higher birth weight was associated with smaller volume. Furthermore, birth weight was positively associated with cerebellar white matter volume in controls, but not ADHD (interaction: *p* = .021). The interaction of genotype with birth weight affecting cerebellum gray matter is consistent with models that emphasize increased influence of genetic risk-factors in an otherwise favorable prenatal environment. The absence of an association between birth weight and cerebellum white matter volume in ADHD suggests that other genetic or environmental effects may be at play, unrelated to XKR4. These results underscore the importance of considering environmental effects in imaging genetics studies.

## Highlights

► Cerebellum changes, XKR4 G-allele, and birth weight (BW) are associated with ADHD. ► We studied effects of XKR4 and BW on brain volumes in ADHD and controls. ► BW was negatively associated cerebellum gray matter in XKR4 G-homozygotes. ► BW was positively associated with cerebellum white matter in controls but not ADHD. ► Results suggest environmental influence should be considered in imaging genetics.

## 1 Introduction

Attention-Deficit/Hyperactivity Disorder (ADHD) is a heritable neurodevelopmental disorder characterized by age-inappropriate symptoms of inattention, hyperactivity and impulsivity (American Psychiatric Association, 2000; Faraone et al., 2005). Structural and functional changes in cerebellum are common findings in ADHD (Durston et al., 2009; Valera et al., 2007), but the genetic and/or environmental risk-factors related to these have not yet been established. Recently, a gene with preferential expression in cerebellum was found to be nominally associated with ADHD: two family-based genetic association studies confirmed a suggestive association of a single-nucleotide polymorphism (SNP) in the XKR4-gene (XK-Kell blood group complex subunit-related family, member 4) (Lantieri et al., 2010; Neale et al., 2008). Although the function of this gene in the brain is not yet entirely clear, it codes for an inferred protein related to the XK-protein, part of the XK-Kell blood group complex (Lee et al., 2000). Whereas Kell and XK proteins are co-expressed in erythroid tissue, this is not the case in non-erythroid tissue (Claperon et al., 2007; Lee et al., 2007). Using a mouse model with later confirmation in human tissue, XK has been found to be overexpressed in the brain in comparison to Kell and shows widespread expression with notable, replicated peaks in the Purkinje cells of the cerebellum (Claperon et al., 2007; Lee et al., 2007). The XK gene has been linked to McLeod syndrome, a syndrome with central nervous system, neuromuscular, and hematologic manifestations in males including movement, cognitive and psychiatric impairments (Danek and Walker, 2005).

Finally, the XKR4-gene itself has been associated with substance abuse (Uhl et al., 2008), and a SNP slightly upstream from XKR4 has been associated with response to antipsychotic medication (Fijal et al., 2012; Lavedan et al., 2009), underscoring its potential relevance to psychiatric phenotypes.

Environmental influences may also be particularly relevant to cerebellum. In previous work in children with ADHD and their unaffected siblings, we have found that familial effects on cerebellum volume were less pronounced than in other brain areas (Durston et al., 2004). In addition, cerebellum is one of the least heritable brain structures at birth (Gilmore et al., 2010) and in childhood (Peper et al., 2007), although other findings suggest that its heritability may increase into adolescence and adulthood (Peper et al., 2009; van Soelen et al., 2011). The cerebellum may be particularly vulnerable to effects of prenatal adversity, as its development begins early in intrauterine life and is markedly protracted (Limperopoulos et al., 2005; Ten Donkelaar et al., 2003; Tiemeier et al., 2010). One measure often used as a proxy for intrauterine conditions is birth weight (Mill and Petronis, 2008). The association of low and very low birth weight (defined as a birth weight < 2500 and < 1500 g respectively) with atypical cognitive and behavioral development is well established (Aarnoudse-Moens et al., 2009; Mick et al., 2002; Nigg and Breslau, 2007; Rice et al., 2007), with a number of neuroimaging studies showing changes in cerebellum in children born with (very) low birth weight (Limperopoulos et al., 2005; Lowe et al., 2011; Parker et al., 2008; Peterson et al., 2000; Shah et al., 2006; Srinivasan et al., 2006; Taylor et al., 2011). However, birth weight may also affect developmental outcome across its full spectrum, as it has also been associated with neurocognitive outcome in the normal range (> 2500 g) (Eriksen et al., 2010; Petersen et al., 2009; Shenkin et al., 2004).

Birth weight is only moderately heritable, with estimates of its heritability ranging from 10 to 40% (Gielen et al., 2008). Greater influences on this measure include an array of environmental factors, including gestational duration, placental dysfunction, maternal body size, nutrition, diseases and infections and maternal use of alcohol or cigarettes during pregnancy (Gluckman et al., 2008; Nigg and Breslau, 2007). Birth weight is thought to affect the development of behavioral problems largely independent of genetic or familial effects (van Os et al., 2001; Wals et al., 2003), where the influence of genetic factors increases with greater birth weight (Wichers et al., 2002). However, the literature on disruptive disorders suggests that there may also be birth weight by genotype interactions in play (Buschgens et al., 2009). Specifically, an interaction between birth weight and dopamine genes (COMT, DAT1, DRD5) has been suggested to affect co-morbid oppositional symptoms in ADHD (Langley et al., 2007; Thapar et al., 2005). Thus, there is evidence for both independent and interactive effects of candidate genes and birth weight in ADHD.

We set out to investigate gene and environmental effects, as well as their interaction, on cerebellum volume in ADHD in an hypothesis-driven design. Specifically, we investigated the effects of XKR4-genotype and birth weight, as both of these are likely to have preferential effects on cerebellum. Given the expression pattern of XKR4 in the brain, we hypothesized that the allele previously found to be overtransmitted in ADHD in genetic association studies would be related to smaller cerebellum volume, but not to the volume of other brain measures. Furthermore, we hypothesized that birth weight would affect cerebellum volume independently of XKR4-genotype. In addition, we investigated whether there were interactive effects of XKR4-genotype and birth weight on cerebellum. Given that earlier results from studies investigating gene/birth weight interactions in ADHD mainly suggested effects on comorbid oppositional symptoms, any results showing an interaction between diagnostic group and birth weight were followed up with analyses testing their specificity to subjects with co-morbid ODD.

## 2 Method

The Medical Ethical Review Board of the University Medical Center Utrecht approved the study and its procedures.

### 2.1 Participants

122 Children (64 controls, 58 children with ADHD) aged between 6.6 and 15.7 years participated in this study. Analyses including data on birth weight were conducted on a subset for whom these data were available (51 controls and 37 children with ADHD). The subjects were enrolled in an ongoing longitudinal cohort study of brain development in ADHD. As such, the dataset consisted of new subjects (69% of the total sample) and subjects who had previously been included in another imaging genetics study (31%, equally distributed between patients and controls) and for whom DAT1 and DRD4 genotype had previously been determined. The previous study did not investigate cerebellum or any genes other than the two mentioned (Durston et al., 2005). The remaining subjects had not been previously genotyped.

Groups were matched for age, gender and IQ at the group level. Demographic and clinical characteristics are given in Table 1. Written informed consent was obtained from parents after full disclosure of the study purpose and procedure. Children provided written and/or verbal assent. The DISC-IV, parent version (Shaffer et al., 2000) was administered to parents in order to confirm the clinical diagnosis of ADHD (ADHD group) or to exclude psychiatric morbidity (controls). The DISC-IV is a widely used standardized structured interview that assesses a broad range of psychiatric diagnoses using DSM-IV criteria. DISC cutoffs for presence/absence of disorders are therefore equivalent to the DSM-IV criteria. Parents filled out the Child Behavior Checklist (CBCL) (Verhulst et al., 1996), to provide a dimensional measure of behavioral symptoms. Controls were excluded if they met criteria for any psychiatric diagnosis or if they had a first-degree relative with a history of psychiatric problems. This was assessed by asking parents for the psychiatric history in the direct family, where a psychiatric diagnosis in a relevant family member was the direct exclusion criterion. Children with ADHD were excluded if they met DISC-IV criteria for a co-morbid disorder other than ODD or CD. In both groups, additional exclusion criteria were any major physical or neurological disorder or the presence of metals in the body that precluded the MRI session. IQ was assessed using a four subtest short form of the Dutch version of the WISC-R or WISC-III (Wechsler, 2005). Information on pregnancy and delivery from a parental questionnaire were available for 88 subjects (51 controls, 37 subjects with ADHD), including information on birth weight, gestational duration, and maternal use of cigarettes and alcohol during pregnancy. Parents (mostly mothers) were asked to give birth weight in grams and gestational duration in weeks. Previous research has shown that such retrospective reports correlate highly with medical records and can reliably be used for group studies (Gadow et al., 2002; Wahlstedt, 2009; Wahlstedt et al., 2009).

### 2.2 Neuroimaging

All subjects participated in an MRI-scan on a 1.5 T scanner (Philips, Best, The Netherlands). The imaging protocol and processing pipeline have been previously reported (De Zeeuw et al., 2012a, 2012b; Durston et al., 2005). Briefly, a T1-weighted three-dimensional (3D) fast field echo scan of the whole head was acquired with 130 to 150 1.5-mm contiguous coronal slices (earlier scans, on Philips Intera; 37 controls and 37 subjects with ADHD) or 160 to 180 1.2-mm contiguous coronal slices (later scans, on Philips Achieva; 27 controls and 21 subjects with ADHD) (echo time [TE] 4.6 ms; repetition time [TR] 30 ms; flip angle 30°; field of view [FOV] 256 mm; in-plane voxel size 1 mm × 1 mm).

All brain scans were coded to ensure rater blindness to subject identity and diagnosis. The T1 images were automatically placed in Talairach orientation (Talairach and Tournoux, 1988) without scaling, by registering them to a model brain in Talairach orientation (Maes et al., 1997). After linear registration to the T1-weighted image, an intracranial segment served as a mask for all further segmentation steps. The T1-weighted images were corrected for field inhomogeneities using the N3 algorithm (Sled et al., 1998). An automatic image-processing pipeline was used to define the volume of total brain, cerebral and cerebellum volume, and gray matter (GM) and white matter (WM) (Schnack et al., 2001a; Schnack et al., 2001b). The gray/white separation algorithm accounted for the effects of partial voluming (Brouwer et al., 2010). Segments for cerebrum and cerebellum were visually checked and edited to ensure an accurate segmentation. Suboptimal scan quality precluded gray/white separation for 4 controls (6.3%, of which 3 were also included in the analyses including birth weight) and 3 children with ADHD (5.2%, of which 2 were also included in the analyses including birth weight). These cases were therefore excluded in analyses on gray and white matter volumes.

We conducted exploratory analyses to assess whether the difference in scan type (change in slice thickness and scanner type) between early and later scans significantly affected the volumetric measures (see Supplementary Material 2). In most part, the results suggest that our findings cannot be attributed to this methodological issue. The scan slice thickness effect appeared greatest for cerebellum white matter, but for the findings remained very similar whether or not the slice thickness covariate was included in the model. This suggests that the group-level results are robust against the effect of scan slice thickness. In addition, the collection of data over 10 years suggests that drift or scanner updates may have affected the measured volumes. However, as these factors affect both control and ADHD groups equally this will lead to a systematic error and is not likely to result false positives in between-group comparisons. In Supplementary Material 2 we report some further descriptive analyses to address these issue.

We conducted exploratory analyses to assess whether the difference in scan type (change in slice thickness and scanner type) between early and later scans significantly affected the volumetric measures (see Supplementary Material 2). In most part, the results suggest that our findings cannot be attributed to this methodological issue. The scan slice thickness effect appeared greatest for cerebellum white matter, but for the findings remained very similar whether or not the slice thickness covariate was included in the model. This suggests that the group-level results are robust against the effect of scan slice thickness. In addition, the collection of data over 10 years suggests that drift or scanner updates may have affected the measured volumes. However, as these factors affect both control and ADHD groups equally this will lead to a systematic error and is not likely to result false positives in between-group comparisons. In Supplementary Material 2 we report some further descriptive analyses to address these issue.

### 2.3 DNA collection and genotyping

DNA was collected using buccal swabs as described previously (Durston et al., 2005). We selected a SNP in the XKR4-gene that showed nominal significance in two independent association studies (Lantieri et al., 2010; Neale et al., 2008): rs2939678. It was genotyped using Applied Biosystems' TaqMan SNP assays on ABI Prism 7900 HT real-time thermocyclers. Call rate was > 95%, and the SNP did not deviate strongly from Hardy-Weinberg (HW) equilibrium in controls.

### 2.4 Statistical analyses

We tested for differences in demographic indices using Chi^2^, independent samples *t*-tests and Mann–Whitney *U*-tests as appropriate. We tested for association with ADHD in our samples by using Chi^2^ tests on allele counts. We then investigated effects of the SNP on volumetric measures using univariate GLM for all volumes separately with age, gender, and scan slice thickness as covariates. The effect of T1 slice thickness on MR-based volumes was systematic and equal for patients and controls. As such, a covariate adequately adjusted the analyses for its effects (De Zeeuw et al., 2012a, 2012b; Langen et al., 2009). There were no differences between groups in the distribution across the two T1 slice thicknesses (*p* = .499 for the full set, *p* = .218 for the subset for which birth weight data was available). Main effects of diagnostic group (control/ADHD), genotype and their interaction were entered into the analysis. If the group by genotype interaction was not significant, this term was removed from the model in order to investigate whether genotype affected the brain volumes regardless of diagnostic status. The XKR4 rs2939678 SNP was recoded to a dichotomous A-carrier versus GG measure, as the number of subjects with the AA genotype was low in both groups (1 control subject, 3 subjects with ADHD).

We also computed an index of birth weight standardized by gestational duration by z-transforming birth weight divided by gestational duration. All analyses were repeated with this measure instead of birth weight, but yielded near-identical results. Therefore, we report only the analyses for birth weight.

We investigated the interactive effects using a univariate GLM approach. For each brain volume, we specified a model with age, gender and scan slice thickness as covariates, main effects of group, genotype and birth weight, all second order interactions and the third order interaction (group × genotype × birth weight). Whereas power to detect third order interaction effects was admittedly limited in the current study, the presence of third order interactions would have significant implications and they were therefore included in the first step of the analytic procedure, for exploratory purposes. If the third order interaction was not significant, it was removed from the model in order to test the presence of second order interactions. If these did not approach significance, they were also dropped from the model in order to investigate main effects. If one or more of the second order interactions approached significance, it was further tested whether these were independent of other second order interactions in the model by dropping the other second order interactions, starting with the interaction with the largest *p*-value. If the amount of variance explained by the model (adjusted R^2^) increased when a second order interaction was removed, this interaction was considered a suppressor term and was not carried forward. As such, the final models reported include either all main effects (in order for the reader to appraise their separate effects), or all main effects and the remaining second or third order effects obtained after the model selection procedure described above. The results section explicitly describes the procedure for each result. We then ran a further multivariate GLM, to further investigate complex effects.

## 3 Results

### 3.1 Association results

Table 2 shows the genotype counts and allele counts per group. The SNP was not statistically associated with ADHD in this relatively small sample.

### 3.2 Genotype by diagnostic group effects on full dataset

We found no interaction between XKR4-genotype and diagnostic group for any of the volumes tested (all *p* > .601). However, we found a main effect of XKR4-genotype on cerebellar white matter, both when the group by genotype interaction was included in the model (*F*(1,108) = 4.21, *p* = .040) and when it was dropped (*F*(1,109) = 4.30, *p* = .038; reduced to *p* = .064 with intracranial volume as an additional covariate). The GG genotype was associated with smaller cerebellar white matter volume across diagnostic groups (A-carriers, M(SD) = 50.2(8.1) ml; GG, M(SD) = 45.9(7.2) ml). There were no other main effects of XKR4-genotype (all *p* > .470).

### 3.3 Interactions with birth weight

These analyses were conducted on the subgroup for whom data on birth weight and gestational duration were available (*n*_control_ = 51, *n*_ADHD_ = 37, comorbid ODD in 16 cases). The diagnostic groups did not differ in birth weight (Table 2), nor was there a difference in mean birth weight between the XKR4 genotype groups.

**Fig. 1:**
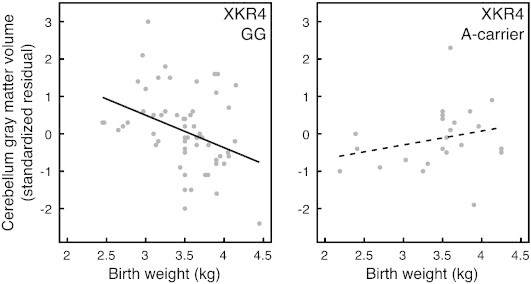
Interaction between XKR4-genotype and birth weight. *n*_control_ = 51, *n*_ADHD_ = 37. Birth weight is negatively correlated with cerebellar gray matter volume in subjects with the XKR4 GG-genotype, regardless of diagnostic group (*r* = − .308, *p* = .017). This relationship is absent in A-carriers (*r* = .272, *p* = .210).

**Fig. 2:**
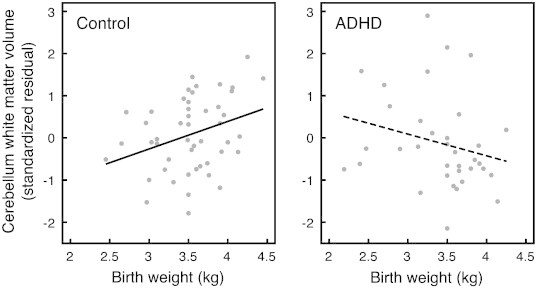
Interaction between diagnostic group and birth weight. *n*_control_ = 51, *n*_ADHD_ = 37. Birth weight is positively correlated with cerebellar white matter volume in controls (*r* = .321, *p* = .026), but not ADHD (*r* = − .240, *p* = .165).

Table 3 shows the results for the analyses of interactions between XKR4-genotype and birth weight. The third order interaction, between diagnostic group, genotype and birth weight did not reach significance for any brain volume. We found an interactive effect of genotype and birth weight on cerebellar gray matter volume and an interactive effect of diagnostic group and birth weight on cerebellar white matter. Figs. 1 and 2 show scatterplots of these results. The full second order model for cerebellum gray matter volume, including all two way interactions between ADHD, XKR4-genotype and birth weight, was suggestive of a genotype by birth weight interaction (*p* = .068). This appeared to be a real and independent effect that was suppressed by other non-significant two-way interactions in the model (group × genotype, *p* = .968 and group × birth weight, *p* = .402), as stepwise removal of these interactions led to statistical significance of the genotype by birth weight interaction (*p* = .031 and *p* = .020 respectively for each removed term), and the variance explained increased with each removal, suggesting that the other two-way interactions were acting as suppressors. In children with the GG genotype, cerebellar gray matter volume was negatively associated with birth weight, regardless of diagnostic group.

In contrast, for cerebellar white matter there were no main or interactive effects of genotype, but there was an interactive effect of diagnostic group and birth weight: In controls only, birth weight was positively associated with cerebellar white matter volume (Fig. 2). The full second order model for cerebellum white matter volume, including all two way interactions between ADHD, XKR4-genotype and birth weight, showed a significant interaction of diagnostic group and birth weight (*p* = .031), but no other significant interactions or main effects. This interaction was independent of the other factors in the model as it retained significance when the other interactions and main effects were consecutively dropped (*p* = .021 when the genotype by birth weight interaction was removed and *p* = .022 when the diagnostic group by genotype interaction was also removed). However, retaining the diagnostic group by genotype interaction in the model resulted in a marginally higher R^2^ than the model without this interaction (R^2^ = .365 and R^2^ = .362 respectively). Therefore we retained this interaction in the model, even though it did not reach significance (*p* = .256, Table 2). The pattern of *p*-values for the main effects was not appreciably different for the models with or without this additional second-order interaction.

When total brain volume was added as a covariate in the analyses of cerebellum, the significance level for the interaction between birth weight and XKR4 genotype for cerebellar gray matter increased (*p* = .004). The significance level for the interaction between diagnostic status and birth weight for cerebellar white matter remained similar (*p* = .021).

### 3.4 Multivariate modeling of cerebellum gray and white matter

To further investigate the complex effects on cerebellum gray and white matter, we modeled these volumes together in a multivariate GLM, including both the diagnosis by birth weight and the genotype by birth weight interactions and total brain volume as a covariate. This model had reduced power compared to the univariate ones, but did permit us to address possible interactions between the results found in the univariate tests. In the multivariate tests, both interactions, as well as the main effect of genotype retained significance (diagnosis by birth weight, *p* = .044; genotype by birth weight, *p* = .012, genotype main effect *p* = .015). The tests for between subject effects showed that a genotype by birth weight interaction was only present for cerebellum gray matter (*p* = .011) but not white matter (*p* = .454). A diagnosis by birth weight effect was only present in cerebellum white matter (*p* = .019, in gray matter *p* = .925). Next to these interaction effects, a main effect of genotype was only found in cerebellum gray matter (*p* = .010), but not white matter (*p* = .624) (Fig. 1). Results for this analysis without the total brain covariate were highly similar with the exception that the multivariate tests for both interaction effects fell short of significance (.05 < *p* < .10), most likely as a result of the reduced power in such a model. At the subsequent univariate level, both interactions retained significance at *p* < .05.

## 4 Discussion

Our results suggest that both XKR4-genotype and birth weight may affect cerebellum volume in ADHD. XKR4-genotype affected the relationship between birth weight and cerebellar gray matter independently of diagnostic group, where subjects with the GG-genotype showed an inverse relationship between birth weight and cerebellar gray matter volume that was absent in carriers of the A-allele. In typically developing children, there was a positive correlation between birth weight and cerebellar white matter volume that was absent in ADHD. This study builds on existing knowledge on cerebellar involvement in ADHD, a replicated finding in neuroimaging studies in ADHD (Durston et al., 2009; Valera et al., 2007). The cerebellum is increasingly understood to be involved in higher cognitive functions such as temporal processing and temporal organization of actions, which are often found to be compromised in ADHD (Durston et al., 2011).

The relationship between XKR4-genotype and cerebellum volume is complex: when investigated in isolation, XKR4-genotype appeared to be associated with cerebellar white matter volume. It was not until it was analyzed with birth weight that its effects on cerebellar gray matter became apparent. An effect on gray matter is more consistent with the expression pattern of the related XK-protein, which is found in gray but not white matter (Claperon et al., 2007; Lee et al., 2007). In subjects with two copies of the allele overtransmitted in ADHD (G-allele) (Lantieri et al., 2010; Neale et al., 2008), there was a negative association between birth weight and cerebellar gray matter volume (Fig. 1). At a first glance, these findings may appear paradoxical. However, cerebellum volume by definition comprises both cerebellum gray and white matter volume, which are statistically mutually dependent as a result. As such, any effect on gray matter can induce a statistical effect on white matter if a relevant moderator is not included in the model. Moreover, the results from the multivariate modeling, where cerebellar gray and white matter are analyzed in tandem, showed main and interactive effects of genotype only for cerebellar gray matter. This suggests that the genotype effect found on cerebellum white matter in the univariate analysis may have been statistically induced by the combination of an effect of genotype on cerebellar gray matter that was moderated by birth weight and the association between birth weight and cerebellar white matter volume in controls. Therefore, we conclude that reductions in cerebellum volume in ADHD (Durston et al., 2009; Valera et al., 2007) may be linked to overtransmission of the G-allele in the presence of higher birth weight. This is consistent with reports suggesting that genetic influences are more likely to affect the phenotype in an otherwise favorable prenatal environment (Wichers et al., 2002). In other words, the hypothesis is that environmental circumstances have higher penetrance to influence cerebellar development when they are adverse. Conversely, genetic risk is hypothesized to higher penetrance in influencing cerebellum development when environmental circumstances are favorable and as such pose less of a restriction.

Importantly, we do not find a G × E interaction operating directly on ADHD. Rather, we find evidence for a G × E interactive effect on a quantitative neurobiological marker of ADHD across diagnostic groups. This is important, as G × E effects on diagnosis per se (dichotomized as control and ADHD-group) are vulnerable to a high false positive rate (Nigg et al., 2010). One way to circumvent this issue is to investigate these effects on a putative intermediate phenotype (such as brain volume). Another approach could be to analyze a continuous measure of ADHD-behavior, which was not adequately possible with our study design (see Supplementary Material 1 for more details).

Importantly, we do not find a G × E interaction operating directly on ADHD. Rather, we find evidence for a G × E interactive effect on a quantitative neurobiological marker of ADHD across diagnostic groups. This is important, as G × E effects on diagnosis per se (dichotomized as control and ADHD-group) are vulnerable to a high false positive rate (Nigg et al., 2010). One way to circumvent this issue is to investigate these effects on a putative intermediate phenotype (such as brain volume). Another approach could be to analyze a continuous measure of ADHD-behavior, which was not adequately possible with our study design (see Supplementary Material 1 for more details).

We found an effect of birth weight on cerebellar white matter volume that was dependent on diagnostic group: in controls, higher birth weight was associated with greater cerebellar white matter volume. This relationship could not be detected in ADHD (Fig. 2). For controls, this result is in line with studies suggesting that cerebellar white matter is preferentially affected in children born preterm (Inder et al., 2005; Limperopoulos et al., 2005; Lowe et al., 2011; Shah et al., 2006; Srinivasan et al., 2006). Of course, a direct comparison with this literature is complicated as the children in this study were not born preterm, or with low birth weight. Nonetheless, this literature does seem to confirm a relationship between birth weight and cerebellar white matter. It is unclear why this relationship was not evident in the ADHD group. Potentially, there may be a genetic effect unrelated to XKR4 operating on cerebellar white matter in ADHD that, similar to our results in cerebellar gray matter, affects cerebellar white matter volume more in children with higher birth weight. However, this is entirely speculative, as no such effect was evident in our data.

The current study is modest in size compared to the large datasets in genetic studies of ADHD. However, it was designed as a hypothesis-driven study to investigate specific gene and environmental effects in an intermediate phenotype related to ADHD (i.e. brain changes), rather than aimed at studying associations between gene and behavior in a more exploratory design. As such, its requirements in terms of sample size are more modest, as a phenotype more proximal to gene expression is being investigated (Durston et al., 2009). That said, the modest sample size may have limited our power to pick up effects, particularly second-order and third-order interactions. In addition, although correction for multiple comparisons is not straightforward in a model building approach analyses, conventional correction methods would render most results short of significance. As such, we acknowledge that our study should be regarded a first step in the direction of G × E imaging studies in ADHD and that replication of these results is warranted in larger samples. A final methodological consideration is that the scans included in this study were collected over a long period of time (roughly 10 years), using the same anatomical protocol, but with two different slice thicknesses. Although we controlled for slice thickness throughout all the analyses, we found that this issue may have had a slightly stronger impact on for the measures of cerebellum white matter (see Supplemental Material 2). Therefore, we recommend that the results for cerebellum white matter be viewed with particular caution.

The current study is modest in size compared to the large datasets in genetic studies of ADHD. However, it was designed as a hypothesis-driven study to investigate specific gene and environmental effects in an intermediate phenotype related to ADHD (i.e. brain changes), rather than aimed at studying associations between gene and behavior in a more exploratory design. As such, its requirements in terms of sample size are more modest, as a phenotype more proximal to gene expression is being investigated (Durston et al., 2009). That said, the modest sample size may have limited our power to pick up effects, particularly second-order and third-order interactions. In addition, although correction for multiple comparisons is not straightforward in a model building approach analyses, conventional correction methods would render most results short of significance. As such, we acknowledge that our study should be regarded a first step in the direction of G × E imaging studies in ADHD and that replication of these results is warranted in larger samples. A final methodological consideration is that the scans included in this study were collected over a long period of time (roughly 10 years), using the same anatomical protocol, but with two different slice thicknesses. Although we controlled for slice thickness throughout all the analyses, we found that this issue may have had a slightly stronger impact on for the measures of cerebellum white matter (see Supplemental Material 2). Therefore, we recommend that the results for cerebellum white matter be viewed with particular caution.

Prenatal exposure to cigarettes and alcohol are important factors that may affect birth weight (Mill and Petronis, 2008; Nigg and Breslau, 2007), and were found in 14–17% of both groups. The numbers were too modest to permit investigation of these factors in the context of the current study, but in a separate, small study, we report that exposure to these substances is indeed associated with smaller cerebellar volumes (De Zeeuw et al., 2012b). The current study suggests that other factors affecting birth weight may be just as important to consider.

As a final consideration, we would like to remind the reader that the XKR4 gene, despite its suggestive association with ADHD and evidence for preferential cerebellar expression is still a largely uncharacterized gene. Much of what we currently know about the gene derives from knowledge on the related XK-gene and XK-protein, a deletion of which leads to McLeod syndrome (Danek and Walker, 2005). Despite the noted expression of XK in the cerebellum, McLeod syndrome has been associated previously with changes in the basal ganglia, a region not addressed in this study. Furthermore, XKR4 does show a scattering of expression outside of the cerebellum (Allen Institute for Brain Science, 2012). This was the motivation for including cerebral volumes as control regions in our study. Finally, SNPs from XKR4 and XKR6 are amongst those that have a high genetic distance between African and European subpopulations, and this may be a factor in differential disease expression across global subpopulations (Hughes et al., 2008). In sum, more work is needed to fully characterize the role of XKR4 in the pathophysiology of ADHD. In a similar fashion, more work is also needed to understand how the cerebellum contributes to the symptoms of the disorder. For example, the relationship of cognitive phenotypes related to cerebellum in ADHD, such as temporal processing (Durston et al., 2011), and brain measures may provide insight in the nature of its involvement in the disorder.

In sum, we show that XKR4-genotype and birth weight both affect cerebellum volume, that some of their effects are interactive and that aspects of these effects differ between ADHD and controls. Furthermore, our results were only fully evident when both genetic and environmental factors were considered. This underscores the importance of considering environmental influences in tandem with genetic effects.

The following are the supplementary materials related to this article.

Supplementary Material 1. An analysis of our data using a dimensional measure of ADHD symptoms rather than diagnosis, showing some issues with to such an approach.

Supplementary Material 2. The effects of scan type/slice thickness on the data.

Supplementary data to this article can be found online at http://dx.doi.org/10.1016/j.nicl.2012.11.010.

## Disclosure

Prof. Durston received a research grant from Unilever Foods which is unrelated to the present study. The other authors report no potential conflicts of interest.

## Figures and Tables

**Table 1:** Demographic characteristics.

		Full dataset with MRI data (*N* = 122)		Subset with both MRI and birth weight data (*N* = 88)	
		Controls (*n* = 64)	ADHD (*n* = 58)	Controls (*n* = 51)	ADHD (*n* = 37)
Gender	N Girls/Boys	9/55	6/52	7/44	5/32
Age	M (SD)	10.1 (1.9)	10.6 (2.1)	10.0 (1.9)	10.6 (1.9)
Total IQ	M (SD)	105.6 (14.9)	103.4 (16.9)	107.4 (15.2)	103.7 (18.3)
Handedness	N Right-/Left-handed/Ambidextrous	56/7/1	46/9/3	43/7/1	29/6/2
DISC-IV	ADHD inattentive		11		9
	ADHD hyperactive/impulsive		11		5
	ADHD combined		36		23
	ODD		22		16
CBCL^a^	Internalising raw score M (SD)	4.7 (3.7)	10.6 (6.1)***	4.8 (3.8)	9.7 (5.8)***
	Externalising raw score M (SD)	5.3 (4.4)	17.8 (9.0)***	5.0 (4.1)	18.1 (9.2)***
	Attention problems M (SD)	2.8 (2.3)	9.3 (2.9)***	2.7 (2.1)	8.8 (3.2)***
TRF^b^	Internalising raw score M (SD)	4.5 (4.8)	7.8 (6.3)**	4.3 (5.1)	8.1 (5.8)**
	Externalising raw score M (SD)	4.5 (5.9)	10.9 (8.9)***	3.7 (5.3)	12.9 (9.2)***
	Attention problems M (SD)	6.8 (6.9)	16.0 (9.3)***	7.0 (7.4)	17.7 (8.9)***
SES	Education father (years)^c^	13.4 (2.7)	12.5 (2.7)	13.4 (2.6)	12.8 (2.5)
Prenatal factors	Birth weight (g) M(SD)			3526 (423)	3447 (565)
	Gestational duration (weeks) M(SD)			39.9 (1.2)	39.4 (1.9)
	Incidence of parent reported smoking during pregnancy^d^			17.6%	17.1%
	Incidence of parent reported alcohol use during pregnancy^e^			14.0%	17.6%

ADHD = Attention-Deficit/Hyperactivity Disorder; ODD = Oppositional Defiant Disorder; DISC-IV = Diagnostic Interview Schedule for Children-Fourth Edition; CBCL = Child Behavior Checklist; TRF = Teacher Report Form; SES = Socio-Economic Status. *p < .05; ** *p* < .01; ***p < .001.

a Unavailable for 2 control and 2 ADHD subjects.

b Unavailable for 9 control and 6 ADHD subjects.

c Unavailable for 6 control and 5 ADHD subjects.

d Unavailable for 3 ADHD subjects.

e Unavailable for 1 control and 3 ADHD subjects.

**Table 2:** Genotyping results.

		Controls (*n* = 64)	ADHD (*n* = 58)	*p*
XKR4 rs2939678	Genotype AA/AG/GG^a^	1/13/50 (0/10/41)	2/19/37 (1/15/21)	.211^b^
	Allele counts (A:G)	15:113	23:93	.080
	Allele frequency (A:G)	11.7%:88.3%	19.8%:80.2%	

ADHD = Attention-Deficit/Hyperactivity Disorder; XKR4 = XK-Kell blood group complex subunit-related family, member 4 gene.

a Genotype frequencies in the subset with data on birthweight are given in parentheses; *p*-values refer to the full dataset.

b Chi-square test should be interpreted with caution since frequency of the AA genotype was low. When dichotomized as A-carrier versus GG, *p* = .080.

**Table 3:** Results for univariate analyses of interactions between birth weight and XKR4 rs2939678.

	Final model	Second order effects			Main effects		
Volume		BW × genotype	Diagnosis × genotype	Diagnosis × BW	BW	Diagnosis	Genotype
Total brain	Main effects only				.168	**.006**	.930
Cerebrum	Main effects only				.146	**.005**	.938
Cerebral Gray	Main effects only				.053	**.017**	.896
Cerebral White	Main effects only				.224	**.043**	.721
Cerebellum total	Main effects only				.173	**.018**	.702
Cerebellum Gray	MEs + BW by genotype	**.020**^a^			.315^b^	.135^b^	**.017**^a^
Cerebellum White	MEs + diagnosis × genotype + diagnosis × BW		.256c,b	**.021**^d^	.853^d^	**.044**^b^	**.044**^b^

ADHD = Attention-Deficit/Hyperactivity Disorder; BW = birth weight; MEs = main effects. The data in this table refer to the sample for which birth weight data was available (*n*_control_ = 51, *n*_ADHD_ = 37) and represents the results from the univariate models. The model selection procedure is described in the results section. Bold typeface indicates significant *p*-values at *α* = .05.

a *p* < .01 when total brain volume was covaried.

b *p* > .05 when total brain volume was covaried.

c Removing this term from the model resulted in less explained variance, which is why it was retained.

d *p* < .05 when total brain volume was covaried.
