# Supplementary material for: Imaging gene and environmental effects on cerebellum in Attention-Deficit/Hyperactivity Disorder and typical development
Source: Neuroimage Clin. 2012 Dec 6;2:103–10. doi: 10.1016/j.nicl.2012.11.010 (PMC3777835; doi:10.1016/j.nicl.2012.11.010)
Supplement: Supplementary Material 1. — An analysis of our data using a dimensional measure of ADHD symptoms rather than diagnosis, showing some issues with to such an approach. [file mmc1.doc]

**SUPPLEMENTARY MATERIAL 1**

***A dimensional analysis***

Multiple authors have brought up the point that the search for GxE interations in psychiatry has a number of possible pitfalls. One of the more important ones concerns using diagnosis as an outcome, dichotomizing a continuous dimension of behavior.^1, 2^ In short, under certain circumstances, using a dichotomous outcome measure will inflate the likelihood of false-positive findings. Thus, in (imaging) genetic and GxE study designs, using a continuous measure of symptoms may be more appropriate. Apart from the conceptual and interpretational issues inherent to studying 3-way interactions between continuous variables (in this case symptoms, environment, brain volume), implementing such a design is not without its own issues. First, what is a valid and reliable measure of symptoms? Ideally, such a measure should be able to sample variance across the normal and abnormal range of behavior. In other words, it should not be heavily skewed, in which case we run the risk of reproducing a dichotomous diagnosis-like situation using a continuous measure (see for example the case of Child Behavior Checklist Attention Problems scale (CBCL-AP) versus the more ecologically valid SWAN rating scale).^3^ Transformation of data using log or square-root transformations is an option, but also complicates interpretation.

Second, most studies in psychiatric disorders are currently designed to compare well described patients, rigorously assessed using standardized research measures, with controls that are equally well assessed not to have any psychiatric disorder. In other words: most studies tend to compare “hypercases” with “hypercontrols”. When continuous measures of any kind are used in these studies, we again run the risk of reproducing a dichotomy since we are analyze a measure with a bimodal distribution. A solution to this problem is sampling across all ranges of attention problems and hyperactivity in the normal population as well as in the psychiatric population. This would require a very different study design and recruitment strategy that is not yet common in studies of psychiatric disorders.

In this supplement, we argue that both problems are in fact present in our study. Despite this situation we do attempt to address two of our main results employing a semi-dimensional account of ADHD symptoms using the CBCL attention problems scale. For this, we use the data for children that had birth weight data available.

The only measure that we have available across participants is the CBCL-AP scale. The CBCL is the best-standardized and most widely used of all psychopathology measures and has proven great clinical utility. However, its use as a continuous measure of behavior problems across controls and patients is problematic since most subscales of the CBCL show a highly skewed distribution in non-psychiatric populations.^3^ Supplementary Figure 1 shows how in our study, a skewed and near bimodal distribution is present in the CBCL-AP ratings.


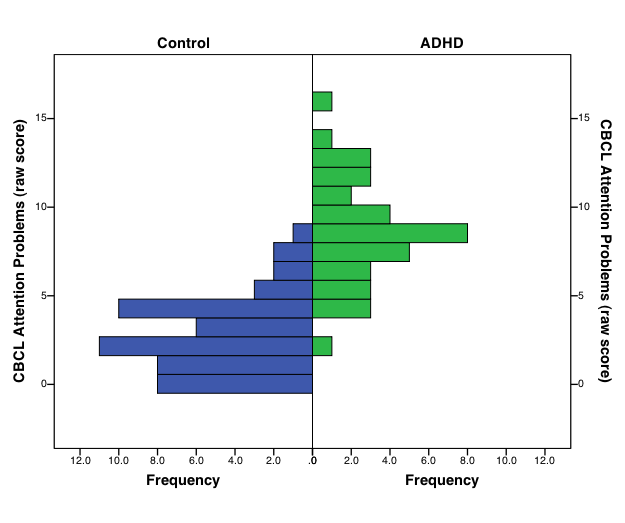


**Supplementary Figure 1.** The CBCL Attention Problems distribution in controls and subjects with ADHD.

*Note.* Only the cases for which birth weight data was available are used here (n_control_ = 51, n_ADHD_ = 37).

CBCL-AP correlates with cerebral volume, and cerebral white matter volume (partial correlations controlling for age, gender and scan slice thickness between r = -.219 and r = -.273, all p < .048) only in the control and ADHD-group combined. As an example, a scatterplot of this relationship for cerebral gray matter volume (Supplementary Figure 3) shows that this is mainly a reproduction of the group effect as the regression line mainly describes the difference between controls and subjects with ADHD as we move along the CBCL-AP scale.


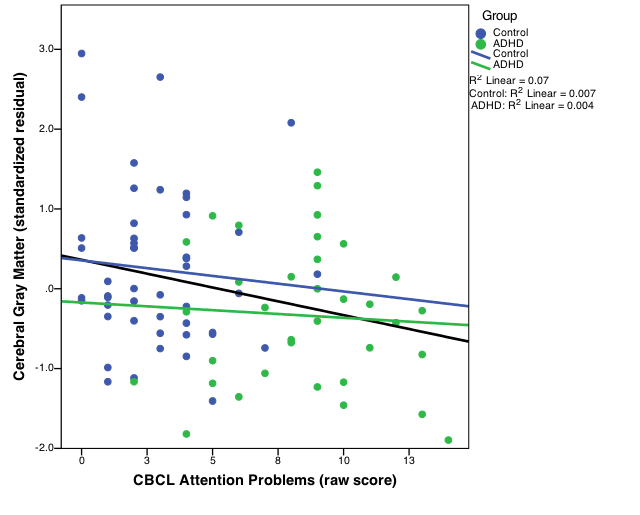


**Supplementary Figure 2.** Scatterplot of cerebral gray matter volume (residualized for the covariates) regressed on CBCL Attention Problems for the entire group (black line), controls (blue) and ADHD-group (green).

*Note.* Only the cases for which birth weight data was available are used here (n_control_ = 51, n_ADHD_ = 37).

One option to normalize the data is to use a square root transformation of CBCL-AP, which translates the data as a whole to a normal distribution, but does not entirely remove the bimodality (Supplementary Figure 3).


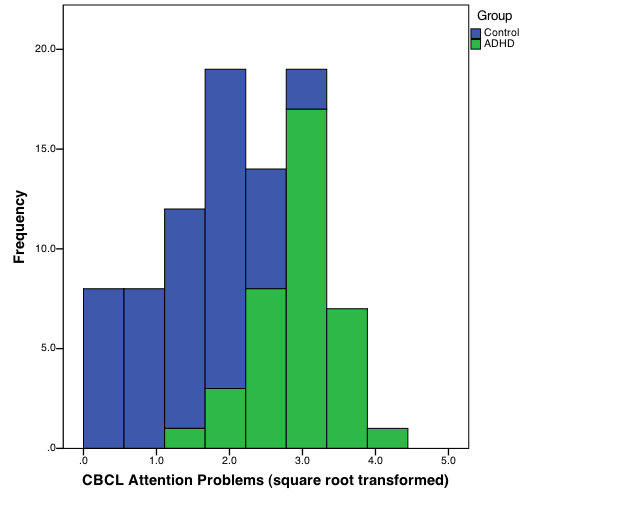

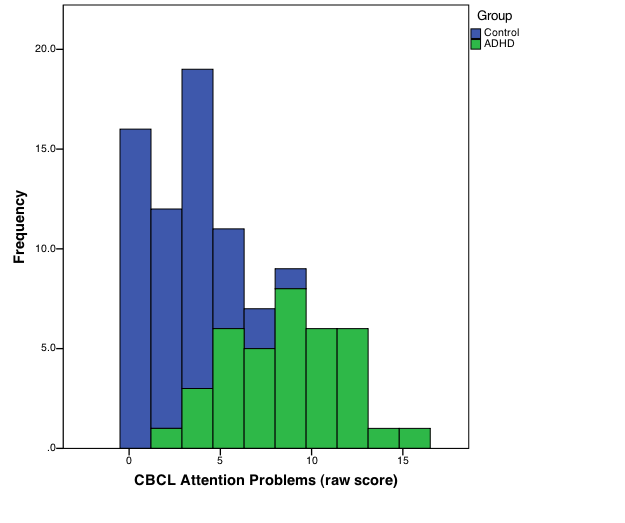


**Supplementary Figure 3.** Stacked histograms of the CBCL Attention Problems distribution for raw data (left) and square root transformed data (right).

*Note.* Only the cases for which birth weight data was available are used here (n_control_ = 51, n_ADHD_ = 37).

Thus, using this latter, square root transformed, data may be more appropriate in standard statistical approaches. Indeed, the (partial) correlations of the square root of CBCL-AP with cerebral volume and cerebral white matter volume are generally somewhat stronger and more significant (partial correlations between r = -.287 and r = -.293, all p < .014). However, the stability of these transformed data in analyses including two other continuous variables is questionable.

We attempted to reproduce the significant group by birth weight interaction that we found for cerebellar white matter using the CBCL-AP scale. In a linear regression model we included the covariates (scan slice thickness, gender and age), birth weight, CBCL-AP and the product of the latter two, the interaction between CBCL-AP and birth weight. We ran two models, using CBCL-AP as is and using the square root transformed data. In both cases, the interaction term did not return significant results (p = .101 for the untransformed data and p = .159 for the transformed data).

We also attempted to visualize the possible dimensional effect of ADHD symptoms using CBCL-AP. We binned the CBCL-AP square root transformed data (Supplementary figure 3, right panel) into 3 bins across all subjects (0-33^th^ percentile, 34-66^th^ percentile, and 67-100^th^ percentile). Because the CBCL-AP is not really continuous in a mathematical scale (i.e., including non-integers), but on a scale of only integers, the bins are not exactly of equal size. Supplementary Figure 4 shows the proportion of controls and subjects with ADHD that fell into the three bins.


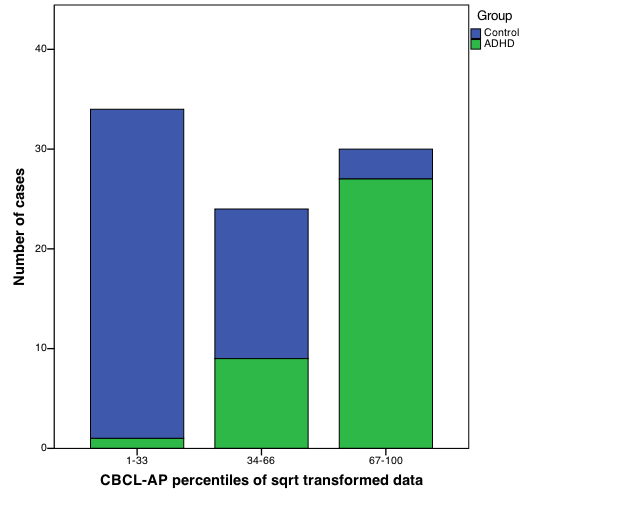


**Supplementary Figure 4.** Stacked histogram of the CBCL Attention Problems percentile bins based on the square root transformed data.

*Note.* Only the cases for which birth weight data was available are used here (n_control_ = 51, n_ADHD_ = 37).

We then plotted cerebellar white matter volume (residualized for the covariates) against birth weight for these three bins separately (Supplementary Figure 5). As can be seen in the Figure, the relationship does follow a graded pattern from the low CBCL-AP group up to the high CBCL-AP group. In none of the three groups however did we find the correlation between cerebellar white matter volume and birth weight (again controlling for the covariates used throughout) statistically significant (0-33 percentile r = .315, p=.101; 34-66^th^ percentile, r = .010, p = .965; 67-100^th^ percentile r = -.146, p=.485).


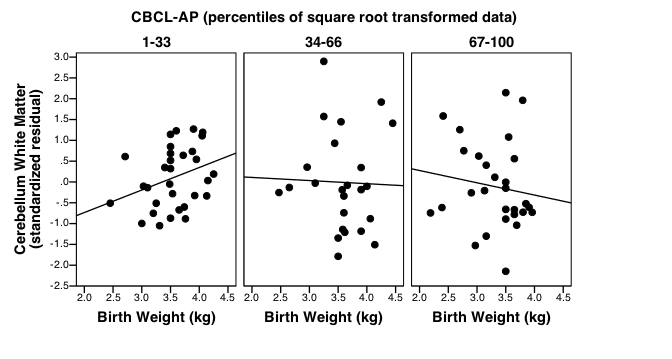


**Supplementary Figure 5.** Scatterplots of Cerebellar White matter (residualized for the covariates used throughout the study) against birth weight for the there CBCL Attention Problems percentile bins.

*Note.* Only the cases for which birth weight data was available are used here (n_control_ = 51, n_ADHD_ = 37).

This pattern of the findings gives us confidence that such graded effects are present and may be more easily picked up when more symptom measures are employed that are more valid across both controls and children with ADHD.

**References**

**1.** Nigg J, Nikolas M, Burt SA. Measured gene-by-environment interaction in relation to attention-deficit/hyperactivity disorder. *J Am Acad Child Adolesc Psychiatry.* 2010;49(9):863-873.

**2.** Eaves LJ. Genotype x Environment interaction in psychopathology: fact or artifact? *Twin Res Hum Genet.* 2006;9(1):1-8.

**3.** Polderman TJ, Derks EM, Hudziak JJ, Verhulst FC, Posthuma D, Boomsma DI. Across the continuum of attention skills: a twin study of the SWAN ADHD rating scale. *J Child Psychol Psychiatry.* 2007;48(11):1080-1087.
