# Supplementary material for: Imaging gene and environmental effects on cerebellum in Attention-Deficit/Hyperactivity Disorder and typical development
Source: Neuroimage Clin. 2012 Dec 6;2:103–10. doi: 10.1016/j.nicl.2012.11.010 (PMC3777835; doi:10.1016/j.nicl.2012.11.010)

**SUPPLEMENTARY MATERIAL 2**

***The effects of scan type/slice thickness on the data***

In order to test the robustness of the results to differences in scan slice, we ran a number of analyses. Here, we used the independent volumes (e.g. cerebral gray, cerebral white, cerebellum gray, cerebellum white) only and used the full dataset (N=122).

**1. Scanner/slice thickness effect versus between group effects**

Scan type was covaried in all analyses of brain volume included in the main paper. We first investigated whether there was a main effect of this variable in any analysis and what its effect size was compared to the effect of diagnosis (i.e., ADHD versus control). The main effect of slice thickness reached significance (p<.001) for cerebellum white matter, but not for any other volume (all p > .21). Figure 1 (below) shows that cerebellum white matter is the only region where slice thickness has an important influence on the estimation of volume.


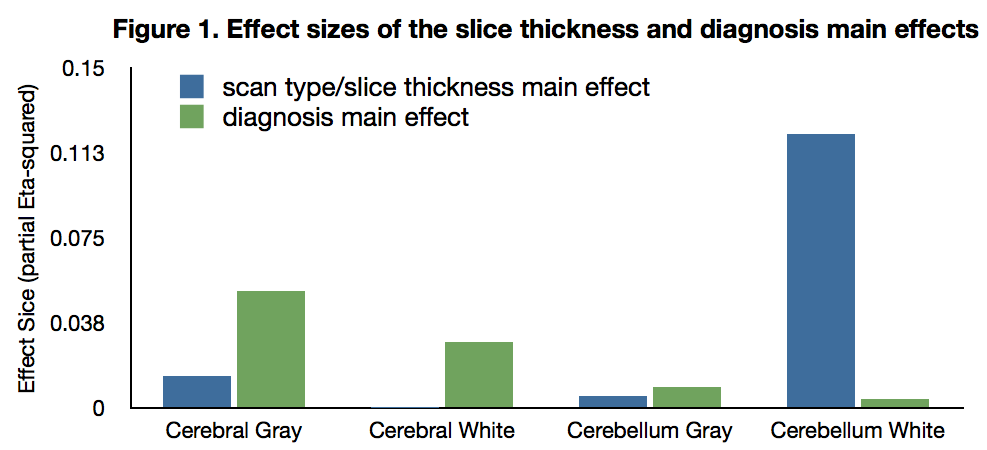


Second, we investigated the effect of in- or exclusion of slice thickness as a covariate in the model on the significance and effect sizes of between group differences on the four regions. For all volumes where the between group comparison (main effect of diagnosis) was significant with slice thickness included as a covariate, this main effect held without the covariate. Figure 2 (below) shows that the effect sizes are also similar. Therefore, even though slice thickness does affect the results to some degree, it does not change the results in terms of between group differences.

**
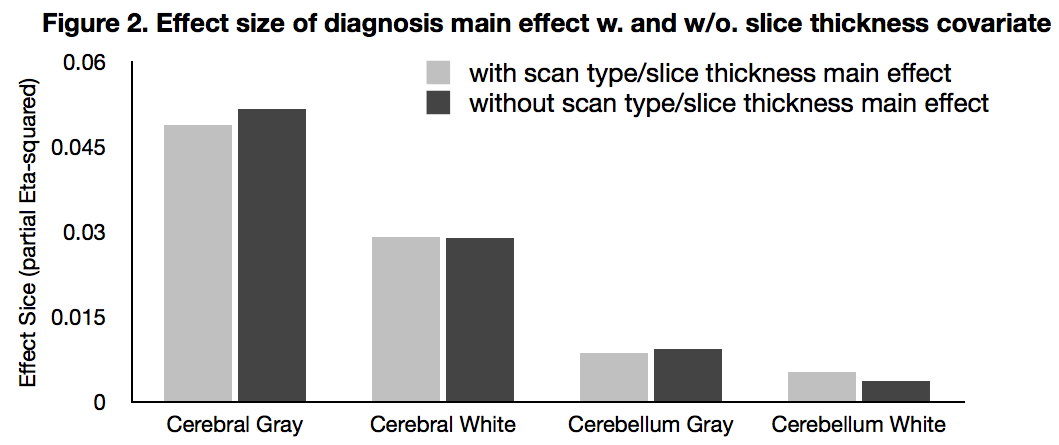
**

Finally, we reran the analysis of the interaction between diagnosis and birth weight from the main paper without the slice thickness covariate. The interaction remained significant without slice thickness as a covariate (p < .01) and the effect size was similar (partial Eta-squared of .093 without the covariate, and .080 with the covariate).

**2. Descriptive analysis of the effect of scanner drift/upgrades across the inclusion period**

We performed descriptive visual inspection of the standardized residuals across the inclusion period of 10 years. We noted in the main paper that a number of scanner updates were conducted during this period and this could have affected the results to some unknown degree. This is an issue that is common to all studies that span significant time frames and are run in clinical setting. Below, we have plotted the standardized residuals for cerebellum gray and white matter as a function of the day since the first scan. These data were residualized for gender, age and scan slice thickness. We chose to also residualize for slice thickness as this was a known change and here we are interested in any patterns in addition to that issue. Figure 3 and 4 (below) clearly show that there is no clearly discernable pattern to suggest that there was systematic drift in the course of the study or that any particular update affected our measures.

**Figure 3. Standardized residual of cerebellum gray matter volume across the study period.**


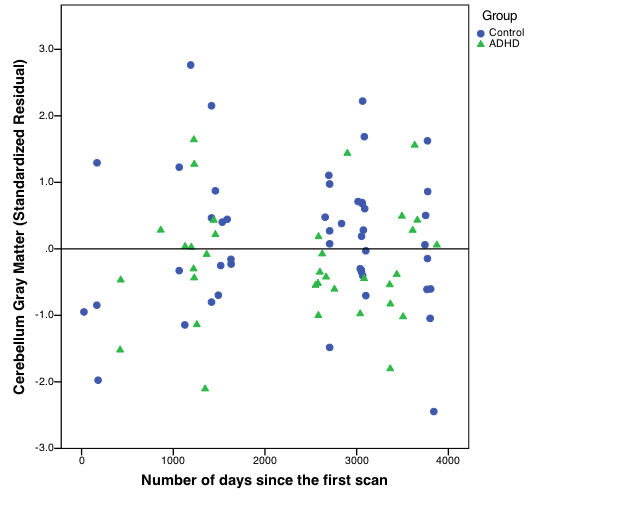


**Figure 4. Standardized residual of cerebellum white matter volume across the study period.**


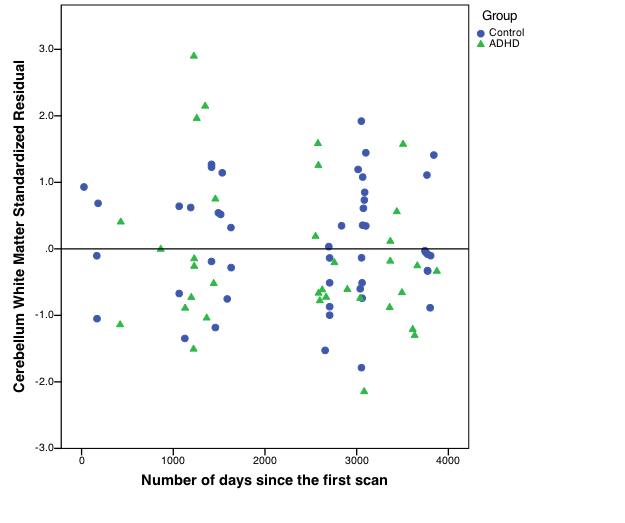

Supplement: Supplementary Material 2. — The effects of scan type/slice thickness on the data. [file mmc2.doc]
